# Activation of Focal Adhesion Pathway by *CIDEA* as Key Regulatory Axis in Lipid Deposition in Goat Intramuscular Precursor Adipocytes

**DOI:** 10.3390/ani15162374

**Published:** 2025-08-13

**Authors:** Peng Shao, Qi Li, Yu Liao, Yong Wang, Yaqiu Lin, Hua Xiang, Zhanyu Du, Changhui Zhang, Jiangjiang Zhu, Lian Huang

**Affiliations:** 1 Qinghai-Tibetan Plateau Animal Genetic Resource Reservation and Utilization Key Laboratory of Sichuan Province, Southwest Minzu University, Chengdu 610225, China; s935246946@163.com (P.S.); liqiligexiao@outlook.com (Q.L.); 19828626311@163.com (Y.L.); wangyong010101@hotmail.com (Y.W.); linyq1999@163.com (Y.L.); xianghua2008411@163.com (H.X.); yuzhan.du@outlook.com (Z.D.); zhangchanghui0074@163.com (C.Z.); zhujiang4656@hotmail.com (J.Z.); 2 Key Laboratory of Qinghai-Tibetan Plateau Animal Genetic Resource Reservation and Utilization, Southwest Minzu University, Ministry of Education, Chengdu 610041, China

**Keywords:** *CIDEA*, intramuscular fat, RNA-seq, focal adhesion pathway, fat deposition

## Abstract

**Simple Summary:**

*CIDEA*, a protein associated with lipid droplets, plays a crucial role in maintaining lipid homeostasis. Our research demonstrates that *CIDEA* increases the levels of lipid droplets and triglycerides while concurrently repressing cell proliferation. Furthermore, we discovered that *CIDEA* facilitates lipid accumulation in goat intramuscular adipocytes through the activation of the focal adhesion pathway. This study enhances our understanding of the molecular processes governing intramuscular fat deposition and provides a theoretical framework that could inform molecular breeding efforts aimed at improving the quality of goat meat.

**Abstract:**

Intramuscular fat (IMF) content determines the quality of goat meat and is regulated by the comprehensive effect of the proliferation and adipogenesis of intramuscular preadipocytes. Our previous RNA-seq data revealed that cell death-inducing DNA fragmentation factor alpha (DFFA)-like effector (CIDE) A was upregulated during the development of intramuscular fat in the longissimus dorsi muscle tissue, implying an important role in lipid homeostasis. However, the mechanism by which *CIDEA*, a member of the CIDE family, regulates intramuscular fat deposition in goat muscle is unknown, so we explored the function and underlying mechanism of *CIDEA* in goat intramuscular preadipocytes. To address this, we altered *CIDEA* in intramuscular preadipocytes and resolved the effect and mechanism of *CIDEA* in adipogenesis through RT-PCR, Western blot, triglyceride and LD determinations, CCK-8, and RNA-seq. It was found that *CIDEA* increased lipid droplets (LDs) and triglyceride contents and inhibited cell proliferation. Meanwhile, the lipid metabolism-related genes *PPARγ*, *C/EBPα*, *SREBP1c*, *PLIN1*, *TIP47*, *ADFP*, *DGAT1*, *ACC*, *FASN*, *ACSL1*, and *FABP3* were upregulated, while the lipolysis and β-oxidation genes *HSL*, *ACOX1*, and *CPT1B*, as well as the proliferation marker gene *CDK1*, were all downregulated upon *CIDEA* overexpression. Differentially expressed genes in *CIDEA* dysregulation groups through RNA-seq were selected and were enriched in the apelin and focal adhesion signaling pathways. Specifically, the Western blot and rescue assays found that focal adhesion, but not apelin, was the key signaling pathway in *CIDEA* regulating lipid deposition in goat intramuscular preadipocytes. In summary, this study reveals that *CIDEA* promotes lipid deposition in intramuscular preadipocytes through the focal adhesion pathway and inhibits cell proliferation. This work clarifies the functional role and downstream signaling pathway of *CIDEA* in intramuscular fat deposition and provides theoretical support for improving meat quality by targeting key phenotype-related genes.

## 1. Introduction

Goat meat is favored by consumers as a high-quality meat food with high protein and low cholesterol [1]. Intramuscular fat (IMF), also known as marbling, plays a crucial role in the tenderness, juiciness, and flavor of meat products, determining the quality of meat [2,3]. The IMF content is controlled by adipocytes’ number and volume, which is regulated by the comprehensive effect of proliferation and adipogenesis processes of intramuscular preadipocytes. Thus, understanding the underlying molecular mechanism of preadipocytes’ proliferation and adipogenesis is essential to understand IMF deposition and, thereby, improve the quality of goat meat.

Cell death-inducing DNA fragmentation factor alpha-like effector A (*CIDEA*), a member of the CIDE gene family, is a lipid droplet-associated protein [4,5], mainly localized on the surface of lipid droplets and the endoplasmic reticulum [6,7]. *CIDEA* plays an important role in lipid droplet expansion and TAG accumulation [8,9]. In mice, *CIDEA*, induced by a high-fat diet (HFD), was highly expressed in brown adipocytes [10,11] and correlated with the development of hepatic steatosis [12,13]. High expression of *CIDEA* showed TAG accumulation in hepatocytes and maintained a healthy obese phenotype in the adipose tissue of transgenic mice [14,15]. Its deficiency resulted in the accumulation of smaller LDs and improved insulin sensitivity in brown adipocytes [6,10] and HFD-induced fatty liver [13], as well as increased whole-body energy expenditure in HFD-fed mice. However, the role and underlying molecular mechanism of *CIDEA* in regulating goat IMF deposition remain to be studied.

Apelin is a peptide hormone identified as the only endogenous ligand for the previously orphaned G protein-coupled APJ receptor [16]. In a previous study, apelin, as an adipokine, inhibited adipogenesis via the mitogen-activated protein kinase (MAPK) pathway [17]. In endothelial cells, apelin promotes cell proliferation by activating the downstream phosphoinositide-3 kinase (PI3K)/Akt signaling pathway [18]. Focal adhesion kinase (FAK) is a key component of the membrane proximal signaling layer in focal adhesion complexes and regulates important cellular processes, including cell migration, proliferation, and survival [19]. By controlling the supply of precursors and the enzyme activity of proteins involved in lipid synthesis, FAK can affect lipid metabolism [20].

This study investigates the effect of *CIDEA* on the adipogenesis of goat intramuscular precursor adipocytes and explores the molecular mechanism of *CIDEA* inducing lipid deposition through the FAK signaling pathway. These data reveal the crucial role of *CIDEA* in the lipid deposition of goat intramuscular preadipocytes, which can help to thoroughly interpret the molecular mechanism of intramuscular fat deposition, thus providing a theoretical basis for the improvement of goat meat quality by molecular breeding.

## 2. Materials and Methods

### 2.1. Ethics Statement

All experimental exercises were isolated and approved by the Institutional Animal Care and Use Committee, Southwest Minzu University (Chengdu, China) (permit number: S2020-013). Tissue-sampling procedures were conducted in strict accordance with the state and institutional animal care and ARRIVE guidelines.

### 2.2. Isolation and Culture of Goat Intramuscular Preadipocytes

Purchased from Sichuan Jianyang Dageda Animal Husbandry Co., Ltd. (Jianyang, China), the 2-day-old Jianzhou big-eared goats were rapidly euthanized via carotid artery bleeding in the laboratory. The longissimus dorsi muscle tissues were collected from 3 male Jianzhou big-eared goats, 2 days old, in a sterile atmosphere, followed by washing with PBS containing 5% penicillin–streptomycin (Boster, PYG0016, Pleasanton, CA, USA) three times. The tissues were minced and then transferred into 50 mL centrifuge tubes. Then, type II collagenase (Sigma-Aldrich, C2-BIOC, St. Louis, MO, USA) was added for digestion at 37 °C for 1.5 h (shaking every 5 min), and digestion was terminated with complete culture medium. The digested tissue was filtered to remove the large undigested tissue. Then, the cell suspension was filtered with a 200-mesh (75 μm) sieve and centrifuged at 2000 rpm for 5 min to obtain the mixed cells. The red blood cell lysate (Boster, AR1118, CA, USA) was used to resuspend the precipitate, followed by standing for 5 min to remove the red cells. Subsequently, the cell suspension underwent centrifugation at 2000 revolutions per minute for a duration of 5 min, followed by resuspension in complete culture medium sourced from Gibco (C11330500BT, Beijing, China). The cells were seeded in a 25 cm^2^ culture plate and cultured at 37 °C in 5% CO_2_. After 2 h, the medium was replaced with fresh complete medium, and the attached cells were considered goat primary intramuscular preadipocytes. All subsequent experiments were performed in three biological replicates and three technical replicates.

The goat primary intramuscular preadipocytes were cultured with the complete culture medium containing 10% fetal bovine serum (12483012, Gibco, Carlsbad, CA, USA) and 90% DMEM/F12 (SH30023−01, Hyclone, Logan, UT, USA) supplemented with 100 U/mL penicillin–streptomycin (080092569, Harbin Pharmaceutical Group, Harbin, China). The medium was changed every two days during this process, and there were no significant changes in the adipocyte morphology. To promote adipogenesis, cells were cultured with an adipogenic medium consisting of complete medium and 50 μM oleic acid (112-80-1, Sigma, MO, USA) for 48 h.

In the FAK pathway inhibition assay, the FAK inhibitor was added at a concentration of 100 nM along with the oleic acid induction solution. The control group was an equal volume of DMSO, and the phenotyping examinations were conducted after adipogenic induction for 48 h.

### 2.3. Goat CIDEA Overexpression Vector Construction and siRNA Synthesis

Based on the CDS sequence of goat *CIDEA* in the NCBI database, the CDS region of the *CIDEA* gene was amplified and inserted into the pcDNA3.1 (+) plasmid, which was double-digested by EcoRI and HindIII, and named the CIDEA-OVER vector. Next, the recombinant plasmid was confirmed through enzyme digestion and DNA sequencing, with the empty pcDNA3.1 (+) plasmid serving as the negative control, designated as pcDNA3.1.

The siRNAs for goat *CIDEA* mRNA were designed and synthesized by Shanghai GenePharma company. siRNA-NC S: UUCUCCGAACGUGUCACGUTT; A: ACGUGACACGUUCGGAGAATT. siRNA-CIDEA393 S: CCACCAUGUACGAGAUGUATT; A: UACAUCUCGUACAUGGUGGTT.

To achieve the overexpression and interference of *CIDEA*, plasmids and siRNAs were transfected into goat primary intramuscular precursor adipocytes in a 6-well plate once the cells reached 80% confluence. These transfections were carried out using Lipofectamine™ 3000 transfection reagent (L3000015, Invitrogen, Carlsbad, CA, USA), following the manufacturer’s guidelines. In addition, 1 μg of plasmids or 120 μM of siRNAs was used for the transfection in each well of the 6-well plate in our research.

### 2.4. Oil Red O Staining and Triglyceride Determination

Two days post-transfection, the relative lipid droplet content was assessed using Oil Red O staining (Solarbio, G1262, Beijing, China). Briefly, cells were fixed with formaldehyde for a 30-min duration. Subsequently, the lipid droplets within the cells were stained with a filtered Oil Red O solution for 20 min following a PBS wash. After three additional PBS washes to remove excess stain, the lipid droplets were visualized and imaged under a microscope. The stained lipid droplets were then dissolved in isopropanol, and their absorbance was quantified at a wavelength of 510 nm.

For the detection of triglyceride content, the cells were rinsed three times with PBS and then incubated with 200 µL of lysis buffer for 10 min at room temperature. The supernatant was heated at 70 °C for 10 min, followed by centrifugation at 2000 rpm for 5 min. The resulting supernatant was then used for triglyceride quantification (Applygen, E1014-105, Beijing, China). The optical density (OD) value of triglycerides was measured at a wavelength of 550 nm. To normalize the triglyceride content, the protein concentration of each sample was determined using the BCA method (Thermo Fisher Scientific, 23225, Beijing, China).

### 2.5. Cell-Counting Kit-8 (CCK-8) Assay

Cells were seeded into a 96-well plate and transfected with *CIDEA* overexpression vectors or siRNAs and their controls individually. After 0, 24, 36, and 48 h of transfection, 10 μL of the CCK-8 reagent (AC11L054, Life-iLab, Shanghai, China) was added to each well. Then, the cells were incubated for 0.5 h at 37 °C. At last, the absorbance was measured using a microplate reader at a 450 nm wavelength.

### 2.6. Western Blot

Cellular proteins were extracted using RIPA buffer (Solarbio Tech Inc., Beijing, China) containing a protease inhibitor (04693132001, Roche, Mannheim, Germany) and a phosphatase inhibitor. The total proteins were then separated via SDS-PAGE electrophoresis and transferred onto PVDF membranes for abundance analysis. This was followed by occlusion using 5% BSA (4240GR100, Biofroxx, Einhausen, Germany), incubation of the primary antibody overnight at 4 °C, TBST washing of the PVDF membrane three times, and 1 h of room-temperature incubation of the secondary antibody. The primary antibodies utilized were anti-β-actin (1:6000, BM0627, BOSTER, Wuhan, China), anti-p-p38-MAPK (1:1000, 3285S, Cell Signaling Technology, Danvers, MA, USA), anti-p38-MAPK (1:1000, 8690S, Cell Signaling Technology), anti-p-FAK (1:1000, ab81298, Abcam, Cambridge, UK), anti-FAK (1:1000, #3285, Cell Signaling Technology), anti-p-AKT (1:2000, 4060, Cell Signaling Technology), and anti-AKT (1:1000, ab32505, Abcam). Goat anti-mouse IgG-HRP (1:6000, BA1050, Boster) and goat anti-rabbit IgG-HRP (1:6000, BA1054, Boster) served as the secondary antibodies. The target proteins were visualized using an enhanced chemiluminescence (ECL) detection system from Thermo (Waltham, MA, USA).

### 2.7. Reverse Transcription–Quantitative PCR (RT-qPCR)

Total RNA was extracted using RNAiso Plus (Takara, Cat. No. 9109), and its concentration and purity were measured. The RNA was then reverse-transcribed using a reverse transcription kit from Vazyme (Cat. No. R32301). RT-qPCR was performed with a Bio-Rad CFX96 PCR System, utilizing the Taq Pro Universal SYBR qPCR Master Mix from Vazyme (Cat. No. Q712-02, Nanjing, China) and gene-specific primers (Table S1). UXT served as the internal reference gene, and the relative expression levels were calculated according to the 2^−ΔΔCT^ method. To clarify the expression pattern of *CIDEA* in goat intramuscular precursor adipocytes, cellular RNA was extracted during the adipogenic induction period (days 0, 2, 4, 6, and 8), and the abundance of *CIDEA* was subsequently detected by RT-qPCR.

### 2.8. RNA Sequencing (RNA-seq)

After 48 h of oleic acid induction, total RNA was extracted using TRIzol (Takara, 9109, Beijing, China) reagent for RNA-seq analysis. The samples were named as the NO group (negative control for overexpression), the AO group (*CIDEA* overexpression), the AS group (*CIDEA* knockdown), and the NS group (*CIDEA* knockdown control). Samples with three replicates were used for high-throughput transcriptome sequencing (Shanghai OE Biotechnology Co, Ltd. Shanghai, China). The Illumina Novaseq 6000 sequencing platform was used to sequence the library and generate 150 bp paired-end reads with 100-300 cycles. DEsq2 was used to screen differentially expressed genes with *p* < 0.05. DEGs were subjected to GO and KEGG analysis.

### 2.9. Statistical Analysis

All experiments were carried out in three biological replicates and repeated three times. GraphPad Prism 9.0 was used for statistical analysis and plotting. Student’s *t*-test and one-way ANOVA were used to calculate the difference. *p* < 0.05 was considered significant, and *p* < 0.01 was highly significant.

## 3. Results

### 3.1. CIDEA Is Associated with Intramuscular Fat Deposition

**Figure 1:**
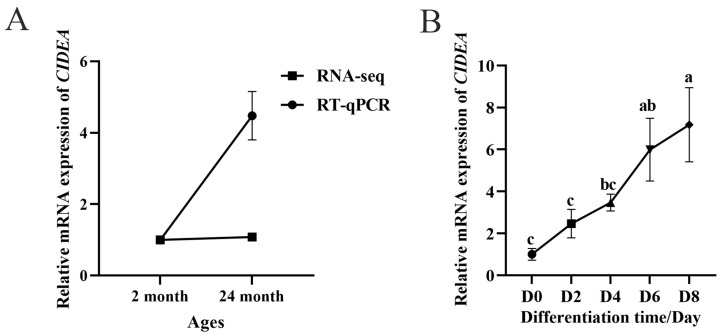
*CIDEA* is associated with intramuscular fat deposition. (**A**) Expression levels of *CIDEA* in longissimus dorsi muscle at different developmental stages (2 months and 24 months). (**B**) Expression pattern of *CIDEA* during differentiation of goat preadipocytes. Distinct lowercase letters signify statistically significant differences (*p* < 0.05).

Our previous RNA-seq data [21] and RNA abundance detected by RT-qPCR showed that the expression of *CIDEA* was upregulated in the longissimus dorsi muscle tissue of 24-month-old goats compared with that of 2-month-old goats (Figure 1A). To further investigate the influence of *CIDEA* on intramuscular adipogenesis, we examined the expression of *CIDEA* during the differentiation of intramuscular preadipocytes in goats. The findings indicated a gradual increase in the expression of *CIDEA* from day 0 to day 8 (Figure 1B), implying that *CIDEA* may play a pivotal role in the adipogenesis of intramuscular preadipocytes.

### 3.2. Overexpression of CIDEA Promotes Lipid Deposition in Goat Primary Intramuscular Preadipocytes

**Figure 2:**
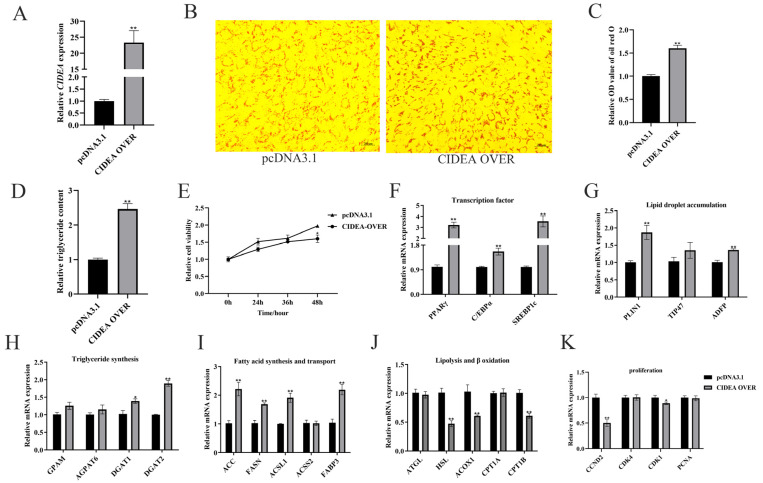
Overexpression of *CIDEA* promotes lipid deposition in goat intramuscular preadipocytes. (**A**) Overexpression efficiency detection. With UXT as the internal reference gene and the negative control as reference. (**B**,**C**) Oil red O staining and quantification of lipid droplets after *CIDEA* overexpression. (**D**) Relative triglyceride content in *CIDEA*-overexpressing cells. (**E**) Cell viability detection after overexpression of *CIDEA*. (**F**–**K**) Effects of *CIDEA* overexpression on the expression levels of genes related to lipid metabolism and cell proliferation. * *p* < 0.05; ** *p* < 0.01.

To elucidate the role of *CIDEA* in lipid deposition in goat intramuscular preadipocytes, we overexpressed *CIDEA* by transfecting the overexpression vector (CIDEA OVER). The results showed that the expression level of *CIDEA* was increased by about 23-fold (*p* < 0.01; Figure 2A). Lipid droplets and TAG contents were both increased after *CIDEA* overexpression (*p* < 0.01; Figure 2B–D). The CCK-8 assay revealed that cell viability was reduced in the CIDEA OVER group (*p* < 0.05; Figure 2E). Correspondingly, the mRNA abundances of transcription factors (*PPARγ*, *C/EBPα*, and *SREBP1c*), lipid droplet accumulation genes (*PLIN1* and *ADFP*), triglyceride synthesis genes (*DGAT1* and *DGAT2*), and fatty acid synthesis and transport genes (*ACC*, *FASN*, *ACSL1*, and *FABP3*) were increased after overexpressing *CIDEA* in intramuscular preadipocytes (Figure 2F–I). However, the mRNA expressions of lipolysis and β-oxidation genes (*HSL*, *ACOX1*, and *CPT1B*) and proliferation genes (*CCND2* and *CDK1*) were both downregulated (Figure 2J,K).

### 3.3. Knockdown of CIDEA Inhibits Adipogenesis in Intramuscular Preadipocytes

**Figure 3:**
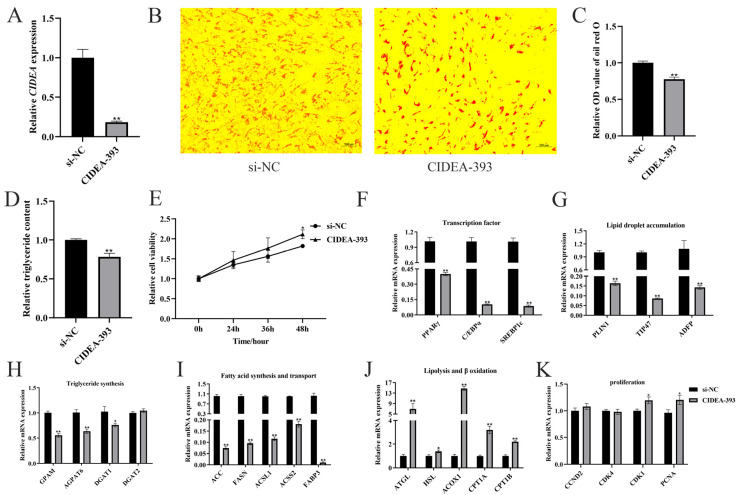
Knockdown of *CIDEA* inhibits adipogenesis of intramuscular preadipocytes. (**A**) Knockout efficiency detection after transfection of CIDEA-393 (siCIDEA) in preadipocytes. (**B**,**C**) Oil red O staining and quantification of lipid droplets content after knockdown with *CIDEA*. (**D**) Relative triglyceride content detection in *CIDEA* knockdown cells. (**E**) Cell viability detection by CCK-8 assay kit after *CIDEA* knockdown. (**F**–**K**) Effect of *CIDEA* knockdown on expression levels of genes related to lipid metabolism and cell proliferation. * *p* < 0.05; ** *p* < 0.01.

Then, we performed siRNA knockdown of *CIDEA* in goat intramuscular preadipocytes, which resulted in a reduction of up to 82% in transcript levels (Figure 3A). Lipid droplets and TAG contents were both decreased in the *CIDEA* knockdown group (*p* < 0.01; Figure 3B–D). The CCK-8 assay showed that siRNA-mediated suppression increased the viability of intramuscular preadipocytes (*p* < 0.05; Figure 3E). Correspondingly, mRNA expressions of transcription factors (*PPARγ*, *C/EBPα*, and *SREBP1c*), lipid droplet accumulation genes (*PLIN1*, *TIP47*, and *ADFP*), triglyceride synthesis genes (*GPAM*, *AGPAT6*, and *DGAT1*), and fatty acid synthesis and transport genes (*ACC*, *FASN*, *ACSL1*, *ACSS2*, and *FABP3*) were all downregulated after knockdown of *CIDEA* in intramuscular preadipocytes (Figure 3F–I). Moreover, the mRNA abundances of lipolysis and β-oxidation genes (*ATGL*, *HSL*, *ACOX1*, *CPT1A*, and *CPT1B*) and proliferation genes (*CDK1* and *PCNA*) were both upregulated (Figure 3J,K).

### 3.4. Screening and Analysis of Differentially Expressed Genes (DEGs) in Intramuscular Preadipocytes with Dysregulated CIDEA Expression

**Figure 4:**
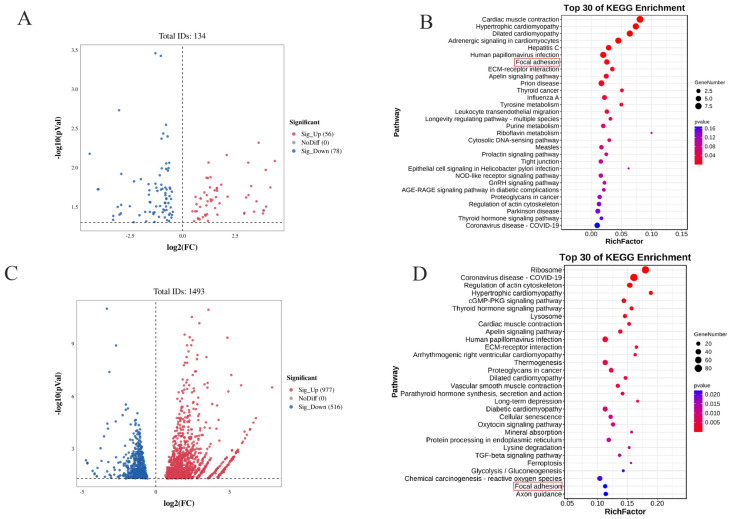
Screening and analysis of differentially expressed genes (DEGs) with dysregulated *CIDEA* expression. (**A**) Volcano plot of DEGs in *CIDEA*-overexpressing precursor adipocytes. Red dots indicate significant upregulation of genes; blue dots indicate significant downregulation of genes. (**B**) KEGG pathway analysis of DEGs in *CIDEA* overexpression group. (**C**) Volcano plot of DEGs in *CIDEA* knockdown precursor adipocytes. Red dots indicate significant upregulation of genes; blue dots indicate significant downregulation of genes. (**D**) KEGG pathway analysis of DEGs in *CIDEA* knockdown group. The red border indicates that the focal adhesion signaling pathway was enriched in the differential genes analysis of transcriptome sequencing results following both overexpression and knockdown of CIDEA. Subsequent experiments confirmed the activation of this pathway.

To elucidate the molecular mechanism by which *CIDEA* regulates lipid deposition in intramuscular preadipocytes, the transcriptional profiles of CIDEA OVER CIDEA-393 groups, together with their controls, were identified by RNA-seq. The RNA-sequencing data and their analysis are listed in Table S2. After *CIDEA* overexpression, we identified 134 differentially expressed genes (DEGs) (*p* < 0.05), of which 56 genes were upregulated and 78 genes were downregulated (Figure 4A). After *CIDEA* expression was reduced in goat intramuscular adipocytes, 1493 DEGs were identified (*p* < 0.05), of which 977 were upregulated and 516 were downregulated (Figure 4C). The heat map revealed that, despite significant differences between groups, the expression patterns were comparable within groups among samples, suggesting minimal variations between individual samples (Figure S2B,D). GO enrichment analysis showed that dysregulated expression of *CIDEA* DEGs was enriched in biological processes related to lipid metabolism, such as C-terminal protein lipidation, fatty acid omega-oxidation, lipid binding, cellular response to lipids, lipid transport involved in lipid storage, and other biological processes associated with lipid metabolism (Figure S2A,C). KEGG pathway analysis indicated that differential mRNAs were involved in apelin and focal adhesion signaling pathways (Figure 4B,D).

### 3.5. CIDEA Regulates Lipid Deposition in Goat Intramuscular Preadipocytes via Focal Adhesion Pathway

**Figure 5:**
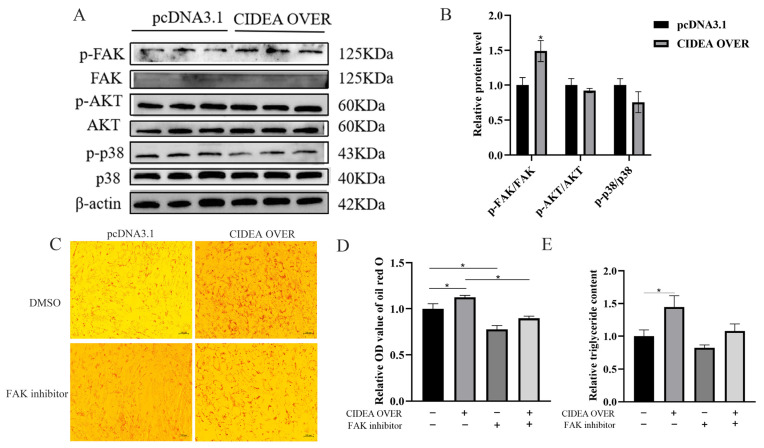
*CIDEA* regulates lipid deposition in goat intramuscular preadipocytes via focal adhesion pathway. (**A**) Detecting protein levels of p-p38, p38, p-AKT, AKT, p-FAK, and FAK after overexpression of *CIDEA* using Western blot. (**B**) Determining ratios of p-FAK/FAK, p-AKT/AKT, and p-p38/p38 upon overexpressing *CIDEA*. (**C**) Lipid droplet content detection after co-transfection of FAK inhibitor or DMSO and CIDEA OVER or pcDNA3.1 by Oil red O staining. (**D**) Determination of relative OD value of lipid droplets extracted after Oil red O staining. (**E**) Intracellular triglyceride content detection after co-transfection of FAK inhibitor or DMSO and CIDEA OVER or pcDNA3.1. * *p* < 0.05.

RNA-seq analysis showed that the apelin and focal adhesion pathways were enriched in both *CIDEA* overexpression and interference cells. This suggests that the effect of *CIDEA* on lipid deposition in goat preadipocytes may be mediated via these two pathways [22]. Therefore, we examined the abundances of the signaling proteins and their phosphorylated forms in these two pathways. The results showed that the abundance of p-FAK and the ratio of p-FAK/FAK were both elevated, while p-38, one key downstream signaling protein of the apelin pathway, exhibited no change (Figure 5A,B). In contrast, knockdown of *CIDEA* decreased the abundances of p-FAK and p-AKT, together with the ratios of p-FAK/FAK and p-AKT/AKT (Figure S4A,B). On this basis, we detected the mRNA expression of FAK and found that overexpression of *CIDEA* promoted FAK mRNA expression, while knockdown of *CIDEA* suppressed its expression (Figure S1A,B). We subsequently employed FAK inhibitors to investigate whether *CIDEA* regulates intramuscular adipogenesis via the focal adhesion pathway [23]. Our findings demonstrated that FAK inhibitors effectively suppressed the focal adhesion pathway (Figure S3A,B). The Oil red O staining results displayed that inhibition of FAK signaling could rescue the lipid droplet content increase induced by *CIDEA* overexpression (Figure 5C,D). The cellular triglyceride content change was consistent with the finding for the lipid droplet content (Figure 5E). Moreover, we found that inhibiting FAK activity further decreased the lipid droplet and TAG contents induced by *CIDEA* interference (Figure S4C,D).

## 4. Discussion

Previous research indicates that *CIDEA* is a pivotal player in both disease pathogenesis and lipid metabolism. Nonetheless, the precise function and regulatory mechanisms of *CIDEA* with regard to intramuscular fat accumulation in goats have yet to be fully understood. Based on our preliminary RNA-seq data, it was observed that the expression level of *CIDEA* was significantly elevated in the longissimus dorsi muscle tissue of 24-month-old goats compared with their 2-month-old counterparts. Consequently, the objective of this study was to explore the impact of *CIDEA* on lipid deposition within goat intramuscular preadipocytes.

Intramuscular fat is mainly determined by adipocyte number and adipocyte volume [24]. Mature adipocytes are unable to divide and differentiate, so IMF deposition is due to the comprehensive effect of progenitor adipocyte cell proliferation and differentiation. Our research shows that the expression of *CIDEA* is upregulated in the longissimus dorsi muscle of 24-month-old goats compared with 2-month-old goats. Since 2-month-old goats are in the juvenile stage, the accumulation of IMF has not yet begun. In contrast, 24-month-old goats have reached adulthood, during which IMF gradually accumulates, leading to a significant increase in its content. This finding aligns with the increase in IMF content reported in our previous study [21]. Despite the different detection methods, the accuracy and depth of the results vary accordingly. Moreover, our study observed a gradual upregulation of *CIDEA* expression from day 0 to day 8 during adipogenic differentiation in goat intramuscular tissue. Analogously, the expression level of *CIDEC*, a homolog of *CIDEA*, was also increased during the differentiation process of human preadipocytes [25]. These findings suggest that *CIDEA* may play a beneficial role in the adipogenesis of intramuscular preadipocytes in goats.

In our study, we observed that the expression levels of several lipid metabolism-related genes were closely associated with *CIDEA*. *DGAT1* and *DGAT2* are rate-limiting enzymes required for triglyceride synthesis [26,27], and our research showed that *CIDEA* promotes the expression of *DGAT1* and *DGAT2* in goat intramuscular adipocytes. *ATGL* and *HSL* are enzymes that facilitate the breakdown of triglycerides into free fatty acids [28,29]. Subsequently, the free fatty acids are transported to mitochondria by CPT1s for β-oxidation [30], or they undergo oxidation by *ACOX1* within peroxisomes [31]. Our research also supports the finding that expression of *CIDEA* leads to the downregulation of a series of lipolysis (*ATGL*, *HSL*, and *ACOX1*) and β-oxidation (*CPT1A* and *CPT1B*) related genes. *FASN* and *ACC*, as the key rate-limiting enzymes in de novo fatty acid synthesis, play a crucial role in lipid production. The upregulation of *CIDEA* expression enhances the expression of these enzymes. Consequently, *CIDEA* promotes lipid deposition by facilitating fatty acid uptake and triglyceride synthesis.

Transcriptional regulatory factors play a significant role in the synthesis of lipids. Previous research has indicated that *CIDEA* acts as an activator of CCAAT/enhancer-binding protein (C/EBP) in the mammary glands of lactating mice [32]. Additionally, the promoter region of *CIDEA* contains a sterol regulatory element (SRE) and peroxisome proliferator-activated receptor (PPAR) elements that can be bound by *SREBP1c* and *PPARγ* [33]. Interestingly, we observed that the expressions of *SREBP1c* and *PPARγ* were affected by *CIDEA* in goat intramuscular preadipocytes (*p* < 0.01), suggesting a potential interaction between *SREBP1c*, *PPARγ*, and *CIDEA*. In dairy goats, *C/EBPα* enhances triacylglycerol synthesis by modulating the activity of the *PPARG* promoter [34]. Our observations are in good agreement with the known effects of *C/EBPα* on *PPARγ*. Other studies have shown that *CIDEA* expression inhibits AMP-activated protein kinase (AMPK) activity, which enhances *PPARγ* expression, thereby increasing triglyceride content, and that *CIDEA* expression promotes the nuclear translocation of *SREBP1c* [35]. Furthermore, expression of *PPARγ* and *SREBP1c* can directly activate the transcription of *FASN* and *ACC*, thereby promoting the formation of lipids [36,37]. On the basis of these results, we hypothesized that *CIDEA* regulated lipid metabolism through *PPARγ* and *SREBP1c*, thereby regulating their downstream genes (*ACC* and *FASN*) expression.

To further unravel the potential molecular mechanism by which *CIDEA* affects IMF deposition in goats, we performed RNA-seq on intramuscular preadipocytes after overexpression and knockdown of *CIDEA*. Interestingly, KEGG pathway enrichment analysis revealed obvious enrichments of apelin and focal adhesion pathways after *CIDEA* dysregulation. It is known that the apelin pathway enhances insulin sensitivity, promotes glucose uptake and utilization, inhibits fatty acid synthesis, and stimulates fatty acid oxidation by activating downstream signaling pathways such as PI3K/Akt and MAPK [38,39,40]. The PI3K-Akt signaling pathway also serves as the downstream signaling of the focal adhesion pathway [23]. In our study, we found that *CIDEA* activated the FAK and AKT signaling proteins, two key signaling proteins in the focal adhesion pathway, but not the p38 signaling protein, a downstream signaling protein of the MAPK pathway, which is consistent with previous findings in mice [41,42]. Intriguingly, our data revealed that interfering with *CIDEA* inhibited the activation of AKT, while overexpression of *CIDEA* did not influence the signaling protein, which needed further exploration. On this basis, we inquired into whether *CIDEA* regulates lipid deposition through the focal adhesion pathway. The FAK inhibitor was used to inhibit FAK signaling. The results suggested that the inhibition of FAK rescued the lipid droplet content increase induced by *CIDEA* overexpression and further decreased the lipid droplet content in *CIDEA*-interfering cells. Unfortunately, due to species limitations, we did not find the antibody matching the goat *CIDEA* protein to characterize *CIDEA* expression at the protein level.

In summary, this study reveals that *CIDEA* promotes lipid deposition in intramuscular preadipocytes through the focal adhesion pathway and inhibits cell proliferation. These works clarify the functional role and downstream signaling pathway of CIDEA in intramuscular fat deposition and provide theoretical support for improving meat quality through manipulating phenotype-related key genes.

## 5. Conclusions

Our study provides evidence for the role of *CIDEA* in intramuscular fat deposition in goats. *CIDEA* promotes adipogenesis through the focal adhesion pathway and inhibits cell proliferation in goat intramuscular preadipocytes. These findings contribute to our understanding of the comprehensive effect of *CIDEA* on intramuscular fat deposition and lay the theoretical foundation for the development of goat molecular breeding technology.

## Data Availability

These RNA-seq data are deposited in the NCBI Sequence Read Archive (SRA) under bio project number PRJNA995405.

## Supplementary Materials

The following supporting information can be downloaded at https://www.mdpi.com/article/10.3390/ani15162374/s1. Figure S1. mRNA abundance detection of FAK in CIDEA-dysregulated intramuscular preadipocytes. Figure S2. Screening and analysis of differentially expressed genes (DEGs) with dysregulated CIDEA expression. Figure S3. Inhibition of focal adhesion pathway by FAK inhibitors. Figure S4. CIDEA regulates lipid deposition in goat intramuscular preadipocytes via focal adhesion pathway. Table S1. Primers for quantitative real-time PCR (RT-qPCR). Table S2. Sequencing data quality control. In addition, the original Western Blot images are available in the Supplementary Materials.
