# Supplementary material for: Activation of Focal Adhesion Pathway by CIDEA as Key Regulatory Axis in Lipid Deposition in Goat Intramuscular Precursor Adipocytes
Source: Animals (Basel). 2025 Aug 13;15(16):2374. doi: 10.3390/ani15162374 (PMC12383094; doi:10.3390/ani15162374)
Supplement: Supplementary file 1 [file animals-15-02374-s001.zip › animals-3735099-supplementary.pdf]

Supplementary Material

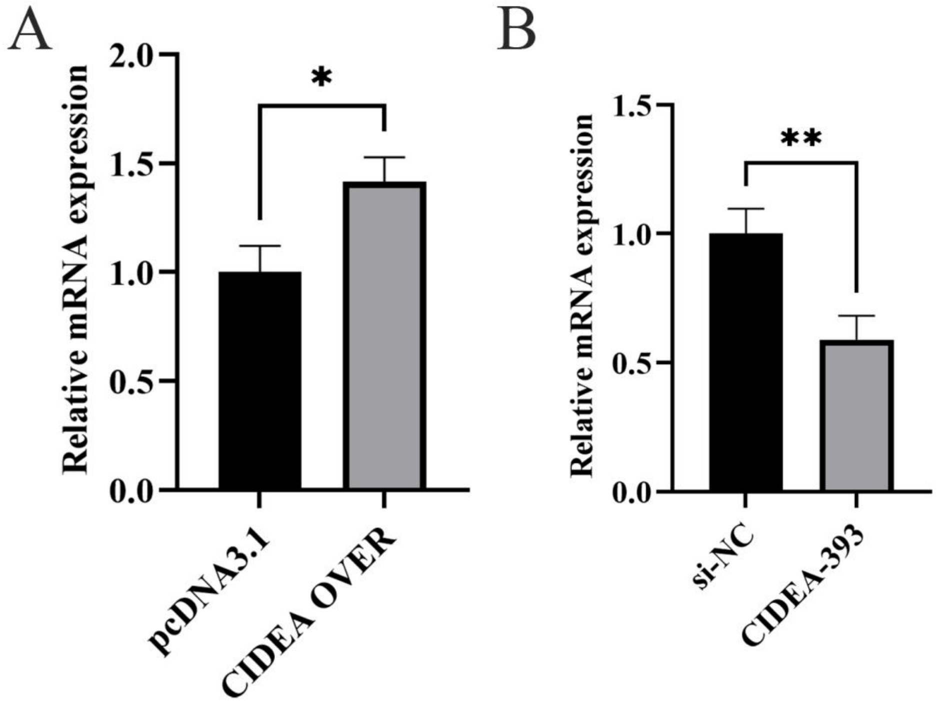

Figure S1. mRNA abundances detection of *FAK* in *CIDEA* dysregulated intramuscular preadipocytes. A. FAK mRNA expression after overexpression of *CIDEA*. B. mRNA expression of *FAK* after knockdown of *CIDEA*.

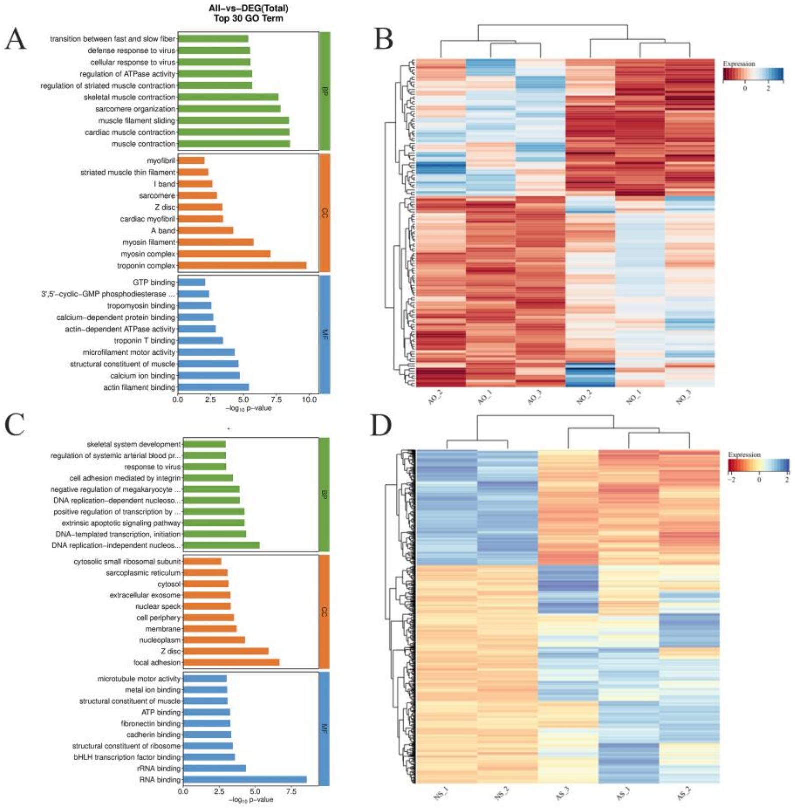

Figure S2. Screening and analysis of differentially expressed genes (DEGs) with dysregulated CIDEA expression

A.GO enrichment analysis of DEGs after *CIDEA* overexpression. B.Heat map of DEGs in *CIDEA* overexpression cells. C. GO enrichment analysis of DEGs after *CIDEA* knockdown. D. Heat map of DEGs in *CIDEA* overexpression cells

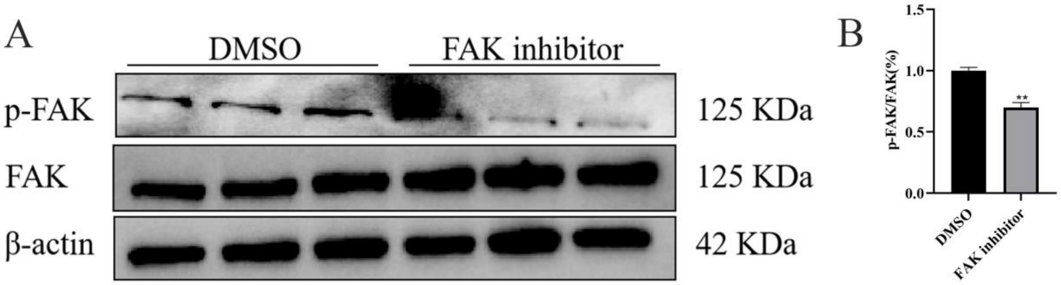

Figure S3. Inhibition of the focal adhesion pathway by FAK inhibitors

A. The protein levels of p-FAK and FAK after FAK inhibitor treatment were detected by Western Blot. B. Measurement of p-FAK/FAK ratio after FAK inhibition

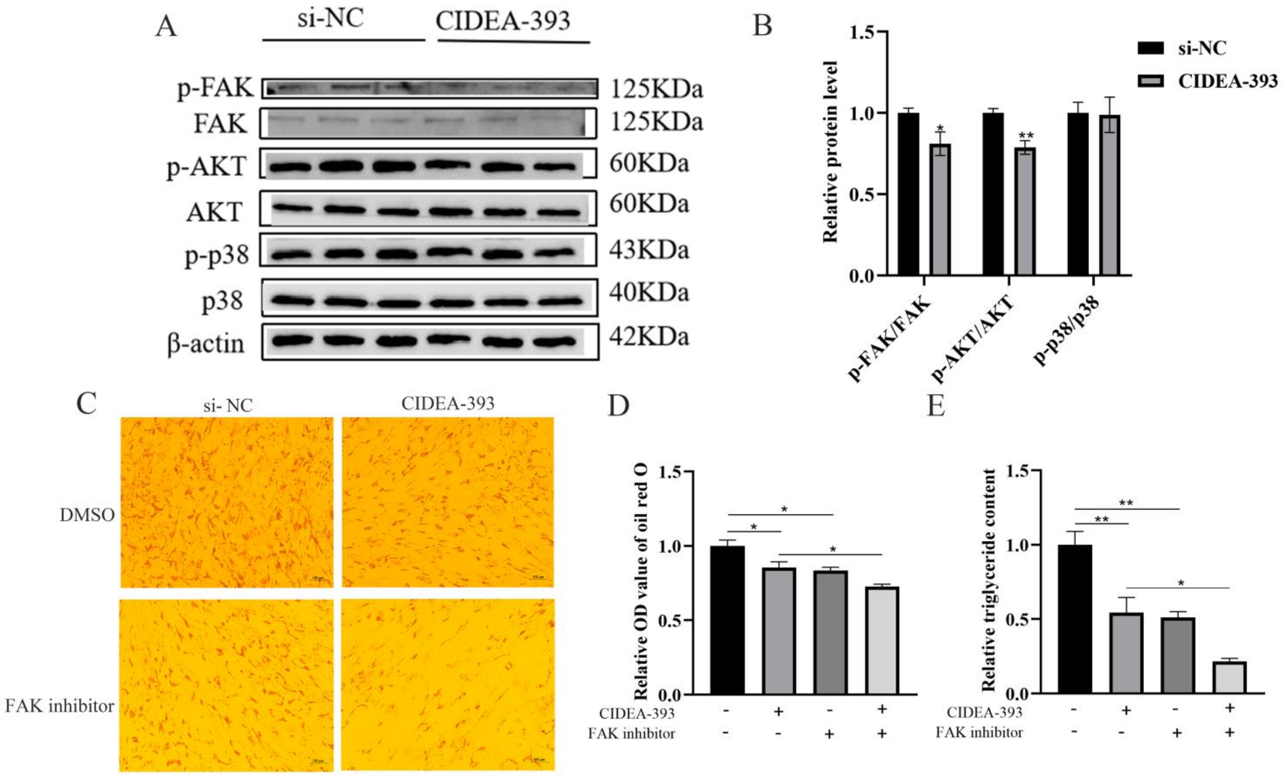

Figure S4. *CIDEA* regulates lipid deposition in goat intramuscular preadipocytes via focal adhesion pathway.

A. Detection of protein levels of p-p38, p38, p-AKT, AKT, p-FAK, and FAK after knockdown of *CIDEA* with Western Blot. B. Determination of ratios of p-FAK/FAK, p-AKT/AKT and p-p38/p38 after interference with *CIDEA*. C. Lipid Droplets content detection after co-transfection of FAK inhibitor or DMSO and CIDEA-393 or si-NC. D. Determination of relative OD value of lipid droplets extracted after Oil red O staining. E. Intracellular triglyceride contents detection after co-transfection of FAK inhibitor or DMSO and CIDEC-393 or si-NC.

Table S1 Primers for quantitative real-time PCR (RT-qPCR)

Gene	Full name	Sequence (5’-3‘)	Tm/°C	Products length/bp	Genbank ID
PPAR γ	Peroxisome proliferator-activated receptor gamma	S: AAGCGTCAGGGTTCCACTATG<br>A: GAACCTGATGGCGTTATGAGAC	60	197	NM_001285658.1
LPL	Lipoprotein lipase	S: TCCTGGAGTGACGGAATCTGT<br>A: GACAGCCAGTCCACCACGAT	60	174	NM_001285607.1
C/EBPα	CCAAT/enhancer binding protein alpha	S: CCGTGGACAAGAACAGCAAC<br>A: AGGCGGTCATTGTCACTGGT	58	142	XM_018062278.1
SREBP1	Sterol regulatory element-binding protein 1	S: AAGTGGTGGGCCTCTCTGA<br>A: GCAGGGGTTTCTCGGACT	58	127	NM_001285755.1
TIP47	Tail-Interacting Protein, 47 KD/Perilipin-3	S:GTCCGCTGACGAGACCGAA<br>A:CAGATTCTCCTCCAGTTTGTCC	60	319	NM_001285595.1
ADFP	Adipose differentiation-related protein/Perilipin 2	S:TTGCTGTTGCCAATACCT<br>A:CTGCATCATCCGACTTCC	60	284	NM_001285596.1
ACC1	Acetyl-CoA carboxylase 1	S: CTCCAACCTCAACCACTACGG<br>A: GGGGAATCACAGAAGCAGCC	60	171	XM_018064174.1
FASN	Fatty acid synthase	S: GGGCTCCACCACCGTGTTCCA<br>A: GCTCTGCTGGGCCTGCAGCTG	66	226	NM_001285629.1
ACSL1	Acyl-CoA synthetase long-chain family member 1	S: TGACTGTTGCTGGAGACTGG<br>A: CAGCCGTCTTTATCCAGAGC	60	220	XM_005698718
ACSS2	Acyl-CoA synthetase short-chain family member 2	S: GGCGAATGCCTCTACTGCTT<br>A: GGCCAATCTTTTCTCTAATCTGCTT	60	100	XM_018057751
FABP3	Fatty acid binding protein 3	S: GATGAGACCACGGCAGATG<br>A: GTCAACTATTTCCCGCACAAG	60	120	NM_001285701
GPAM	Glycerol-3-phosphate acyltransferase, mitochondrial	S: GCAGGTTTATCCAGTATGGCATT<br>A: GGACTGATATCTTCCTGATCATCTTG	60	64	XM_013975269
AGPAT6	Glycerol-3-Phosphate Acyltransferase 4	A: AAGCAAGTTGCCCATCCTCA<br>S: AAACTGTGGCTCCAATTTCGA	60	101	JI861797.1
LPIN1	Perilipin 1	S: CCCATTGCCAGCACTTCAGA<br>A: GCAGCGTACTCGGCAGTATCTC	60	95	XM_018066567.1
DGAT1	Diacylglycerol O-acyltransferase 1	S: CCACTGGGACCTGAGGTGTC<br>A: GCATCACCACACACCAATTCA	60	111	XM_018058729
DGAT2	Diacylglycerol O-acyltransferase 2	S: CATGTACACATTCTGCACCGATT<br>A: TGACCTCCTGCCACCTTTCT	60	100	NM_001314305
ATGL	Adipose triglyceride lipase	S: GGAGCTTATCCAGGCCAATG<br>A: TGCGGGCAGATGTCACTCT	60	178	NM_001285739
HSL	Hormone-sensitive lipase	S: TGCCCAAGACAGAGCCAATG<br>A: GCGGAGGAGCCGAGTATCT	60	181	EU273879.1
ACOX1	Acyl-CoA oxidase 1	S: CGAGTTCATTCTCAACAGTCCT<br>A: GCATCTTCAAGTAGCCATTATCC	60	211	XM_018063771
CPT1A	Carnitine palmitoyltransferase 1A	S: TGACGGCTCTGGCACAAGAT<br>A: CGCGAAGTAGTTGCTATTCAC	60	164	XM_018043311
CPT1B	Carnitine palmitoyltransferase 1B	S: ACGAGGAGTCTCACCACTACG<br>A: GTGTGAAGGACTTGTCGAACCA	60	111	XM_018048994
CCND2	Cyclin D2	S: GGGCAAGTTGAAATGGAA<br>A: TCATCGACGGCGGGTAC	60	173	XM_005680985.3
CDK4	Cyclin dependent kinase 4	S: AAGTGGTGGGACAGTCAAGC	60	199	XM_005680266.3

		A: ACAGAAGAGAGGCTTTCGACG			
CDK1	Cyclin dependent kinase 1	S: AGATTTTGGCCTTGCCAGAG<br>A: AGCTGACCCCAGCAATACTT	60	103	XM_005699058.3
PCNA	Proliferating cell nuclear antigen	S: ATCAGCTCAAGTGGCGTGAA<br>A: TGCCAAGGTGTCCGCATTAT	60	213	XM_005688167.3
UXT	Ubiquitously expressed prefoldin like chaperone	S: GCAAGTGGATTTGGGCTGTAAC<br>A: ATGGAGTCCTTGGTGAGGTTGT	60	180	XM_005700842.2
FAK	Focal adhesion kinase	S:TTGTGCACAGGGACATTGCT<br>A:ATGCGCCCGATCACATCATT	60	280	XM_018058634.1

S: Sense primer; A: Antisense primer.

Table S2 Sequencing data quality control

Sample	Before filtering		Before filtering					Total mapped reads	Multiple mapped	Uniquely mapped
	Raw Reads	Raw Bases	Clean Reads	Clean Bases	Valid Bases	Q30	GC			
AO_1	48.14M	7.22G	47.25M	6.86G	95.03%	94.87%	51.31%	98.50%	4.59%	93.91%
AO_2	51.11M	7.67G	50.16M	7.25G	94.56%	94.87%	52.17%	98.35%	4.79%	93.56%
AO_3	51.64M	7.75G	50.63M	7.37G	95.10%	94.57%	51.60%	98.46%	4.56%	93.89%
NO_1	49.76M	7.46G	48.76M	7.09G	94.97%	94.21%	52.34%	98.18%	5.16%	93.01%
NO_2	47.40M	7.11G	46.53M	6.79G	95.54%	94.94%	51.49%	98.43%	4.62%	93.81%
NO_3	50.94M	7.64G	50.01M	7.24G	94.81%	94.62%	51.74%	98.29%	4.69%	93.60%
NS_1	49.23M	7.38G	48.00M	6.91G	93.54%	94.55%	52.42%	98.52%	5.90%	92.63%
NS_2	49.17M	7.38G	47.98M	6.88G	93.30%	94.95%	52.18%	98.54%	5.82%	92.72%
AS_1	47.66M	7.15G	46.49M	6.70G	93.74%	94.67%	51.21%	98.54%	4.97%	93.56%
AS_2	49.76M	7.46G	48.57M	6.99G	93.64%	95.12%	51.24%	98.61%	4.96%	93.65%
AS_3	49.06M	7.36G	47.76M	7.08G	96.21%	94.67%	50.75%	98.41%	4.68%	93.73%
NS_1	49.23M	7.38G	48.00M	6.91G	93.54%	94.55%	52.42%	98.52%	5.90%	92.63%
