# Supplementary material for: Activation of Focal Adhesion Pathway by CIDEA as Key Regulatory Axis in Lipid Deposition in Goat Intramuscular Precursor Adipocytes
Source: Animals (Basel). 2025 Aug 13;15(16):2374. doi: 10.3390/ani15162374 (PMC12383094; doi:10.3390/ani15162374)

Original Western Blot Images

The original, uncropped and unadjusted western blot images of each protein in our manuscript are displayed as follows. Owing to the multiple proteins needed to be detected in the experiments and saving antibody, we did not incubate the whole membranes.

Figure 5A

p-FAK

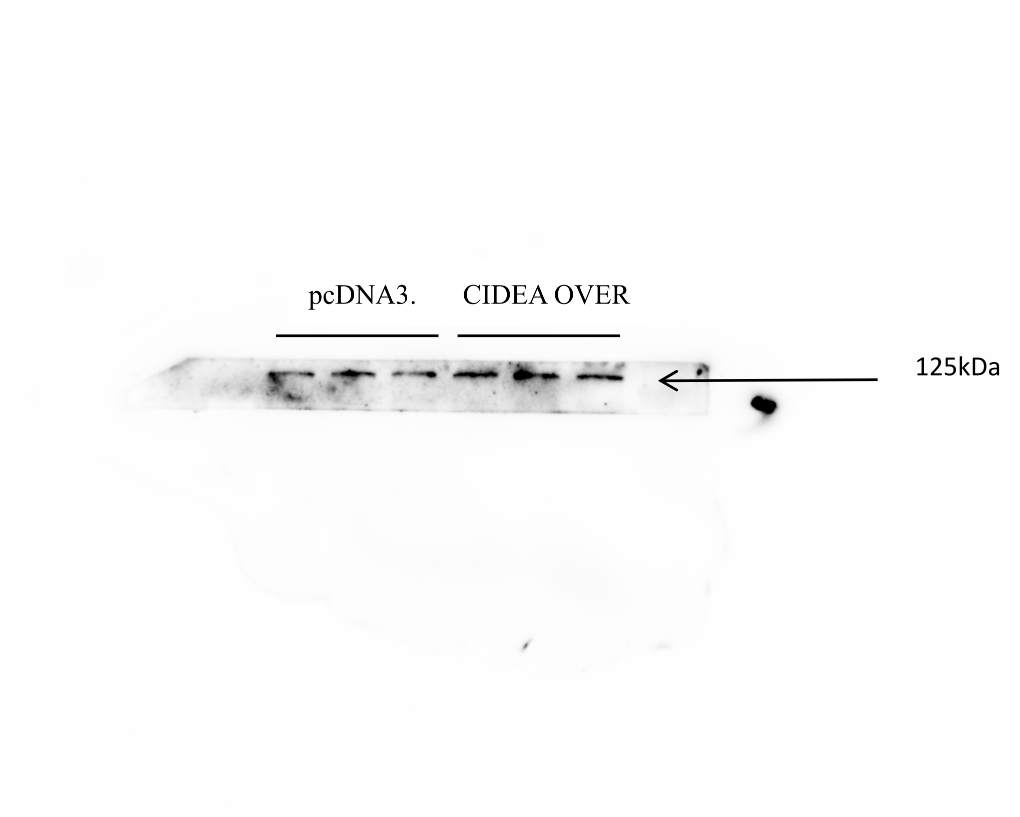

Figure 5A

FAK

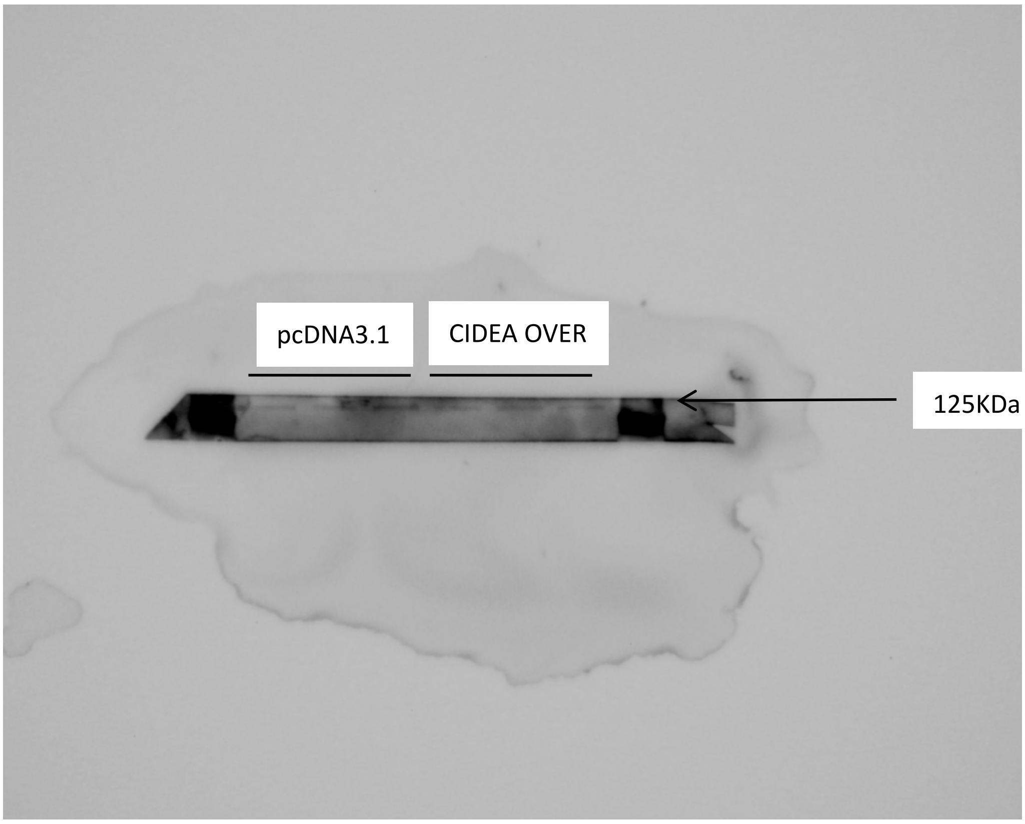

Figure 5A
p-AKT

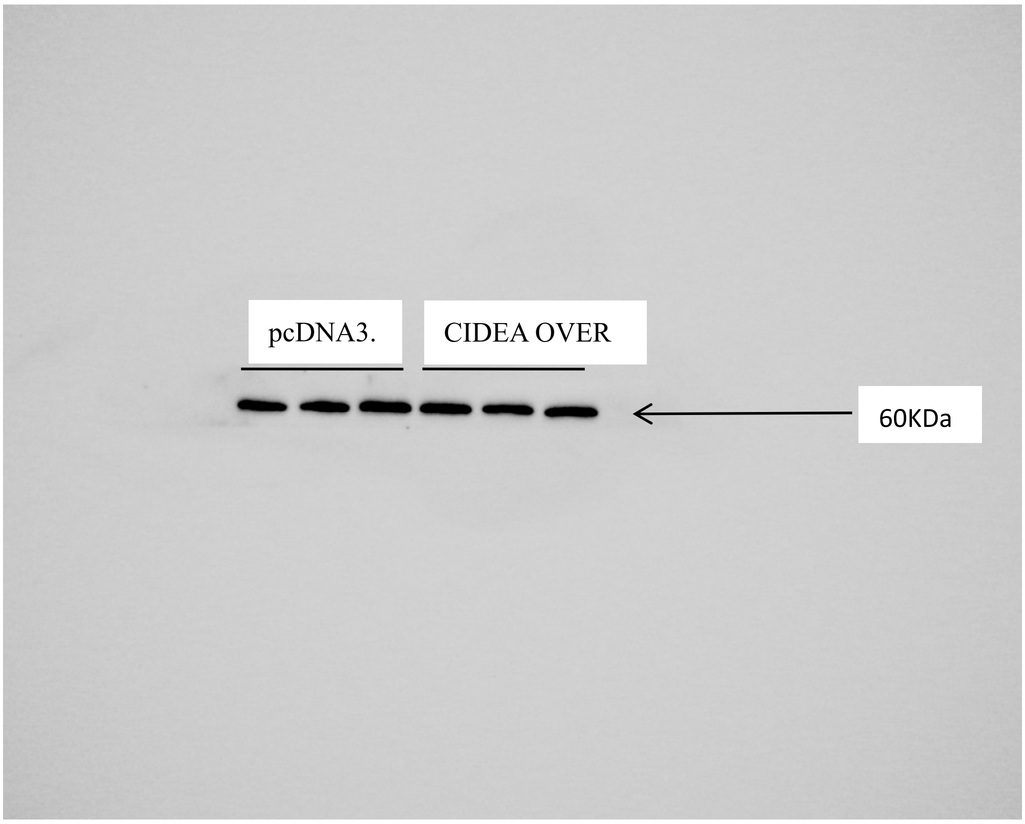

Figure 5A
AKT

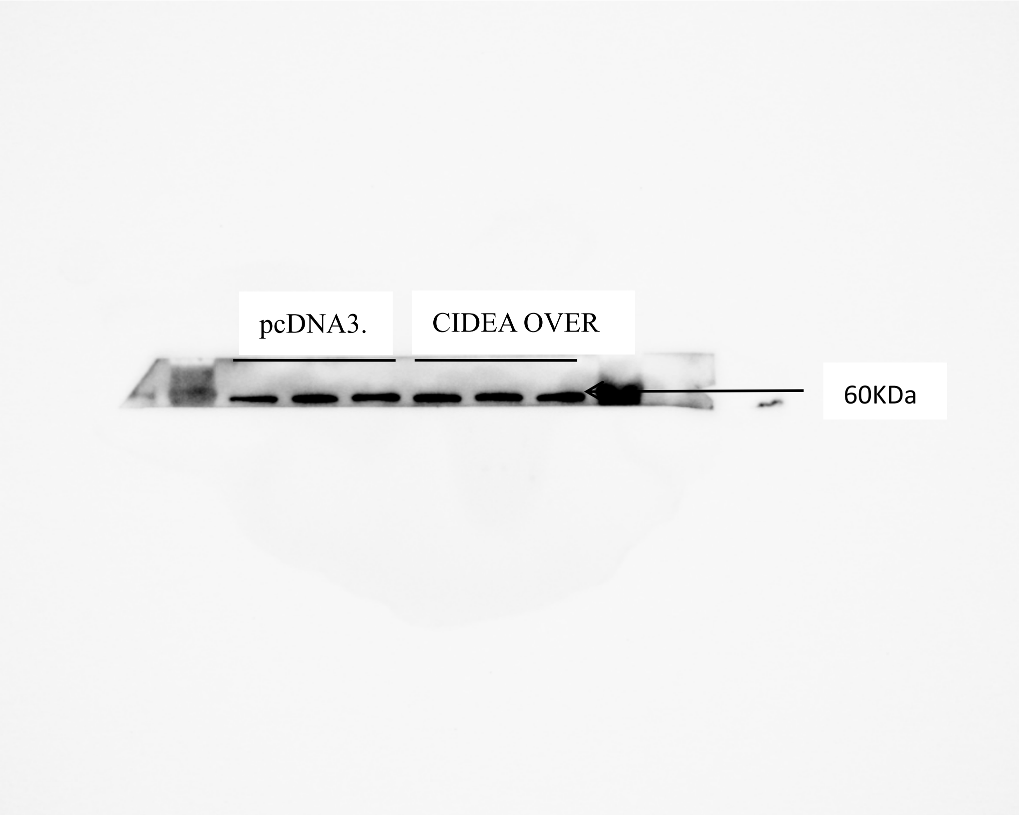

Figure 5A
p-p38

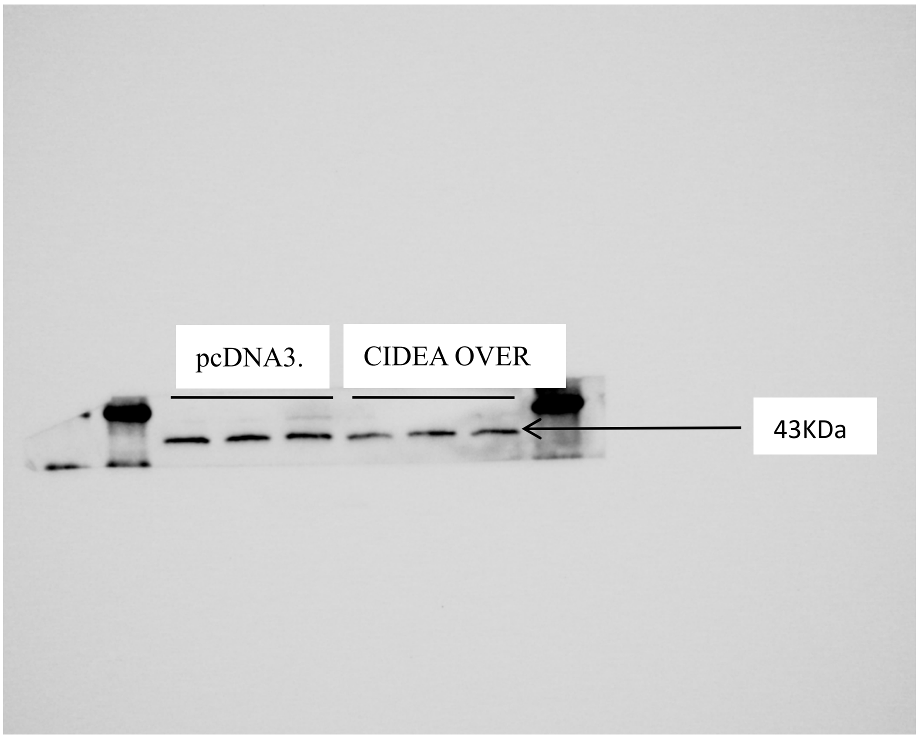

Figure 5A
p38

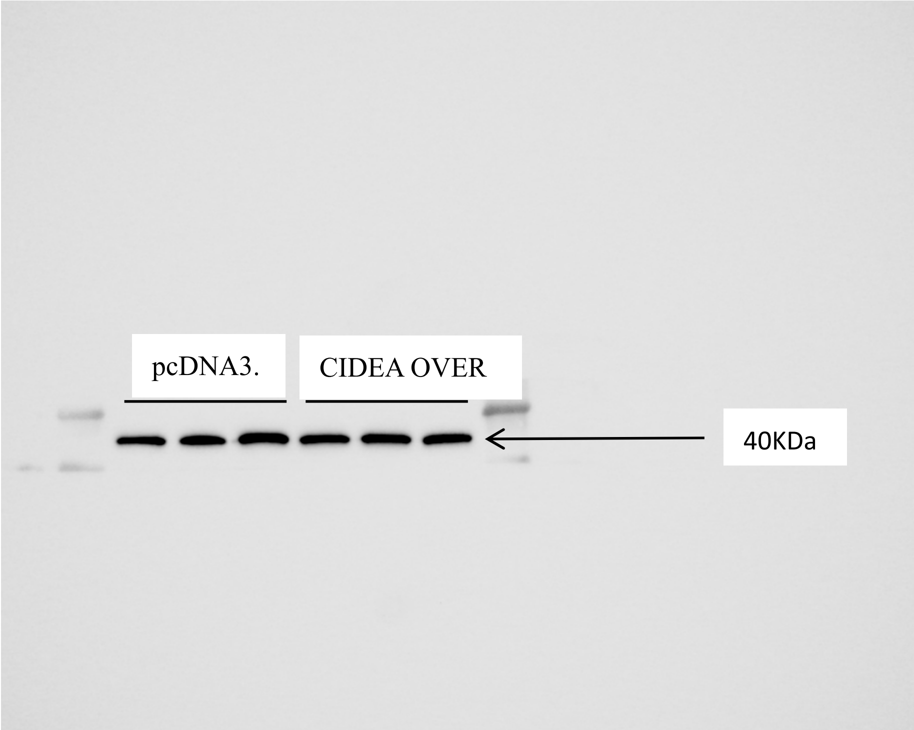

Figure 5A

β-actin

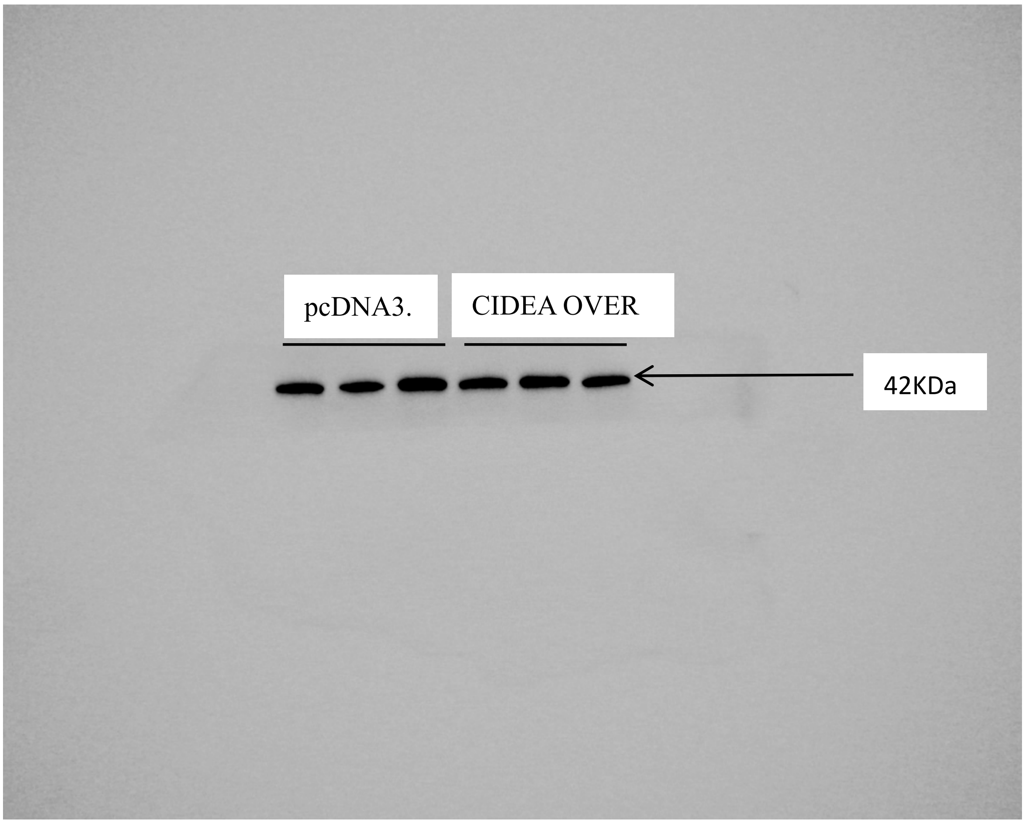

Figure S4

p-FAK

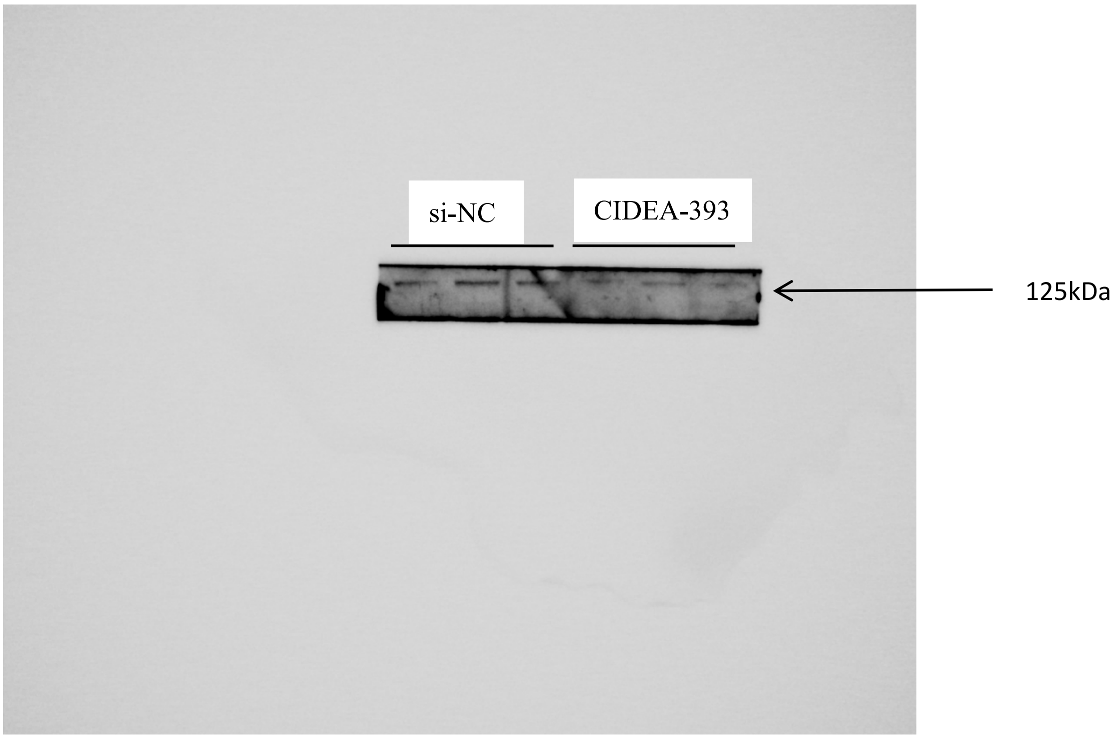

Figure S4

FAK

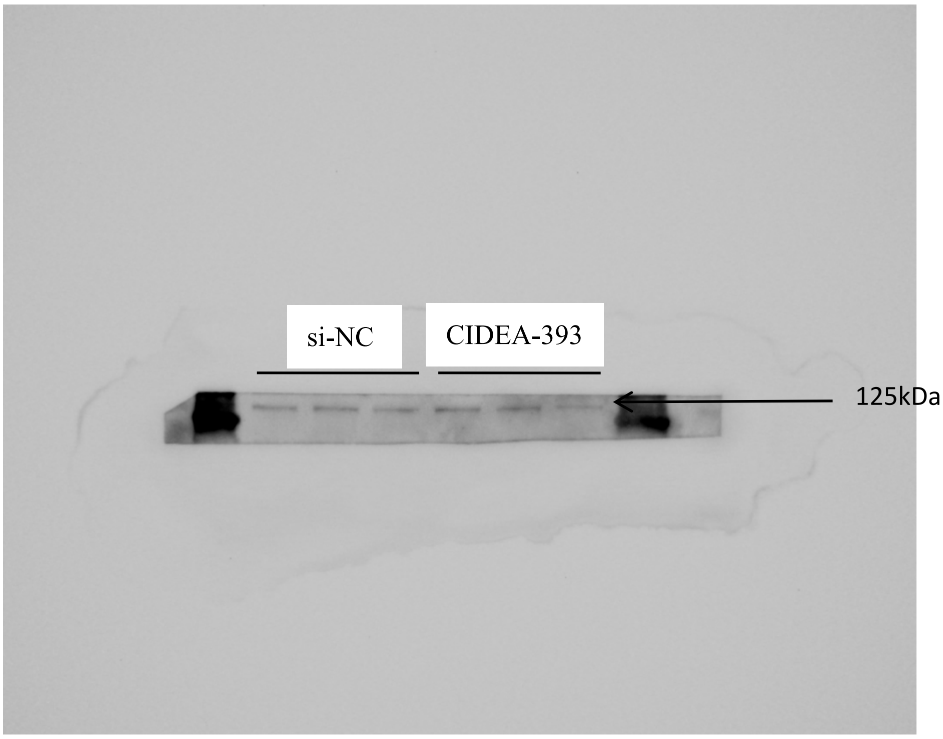

Figure S4

p-AKT

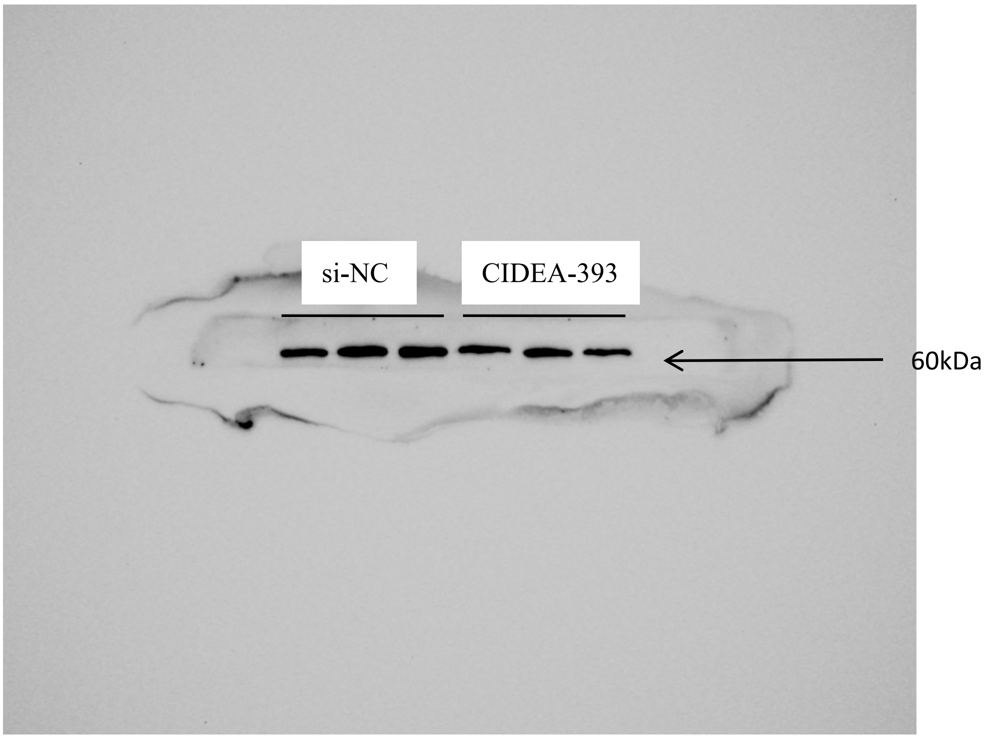

Figure S4
AKT

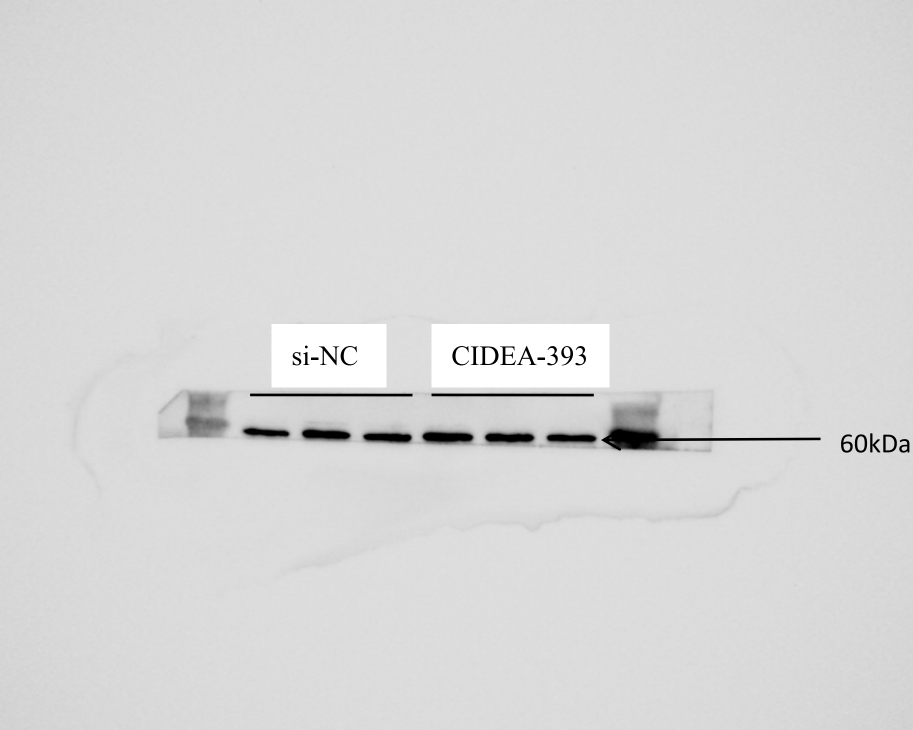

Figure S4
p-p38

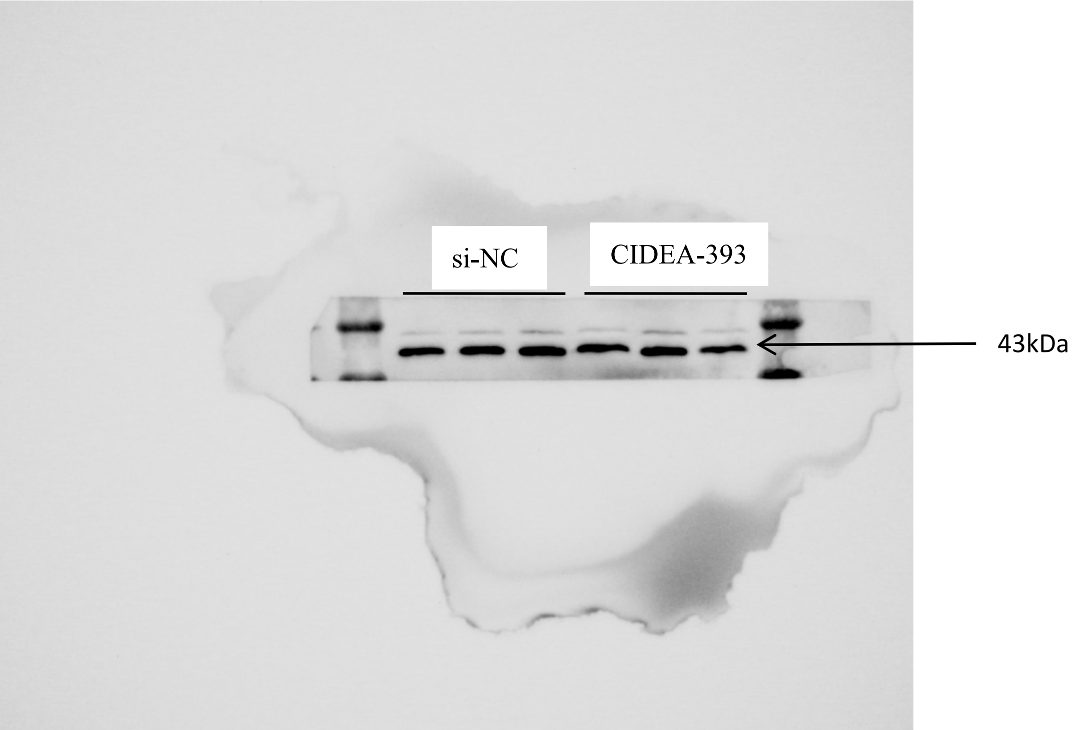

Figure S4
p38

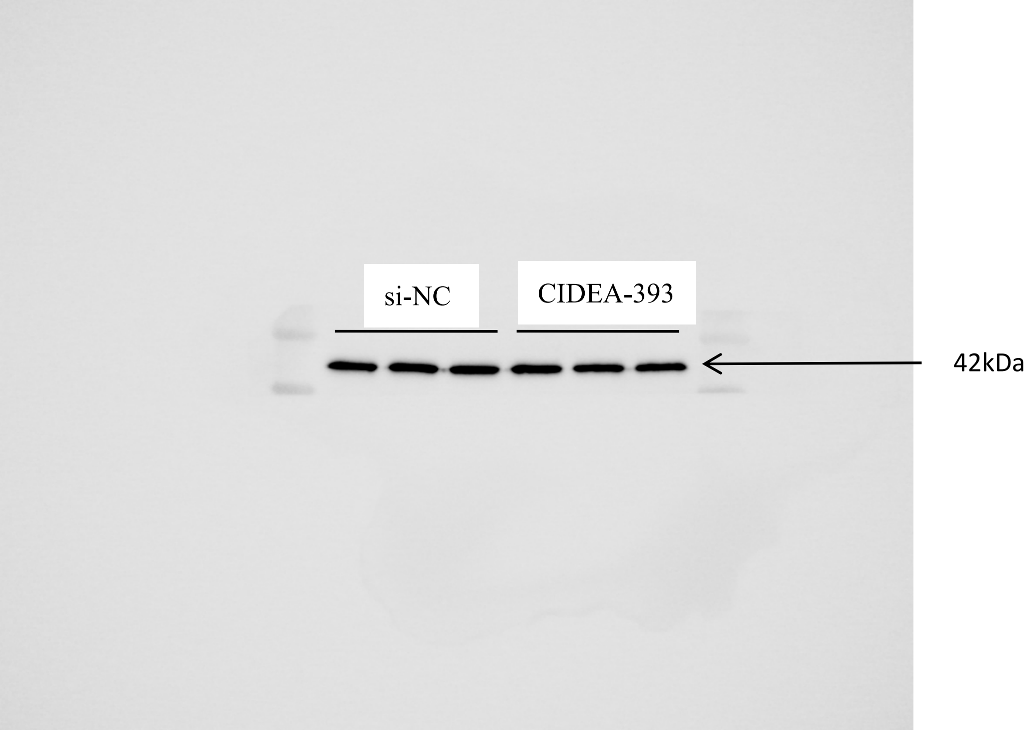

Figure S4
β-actin

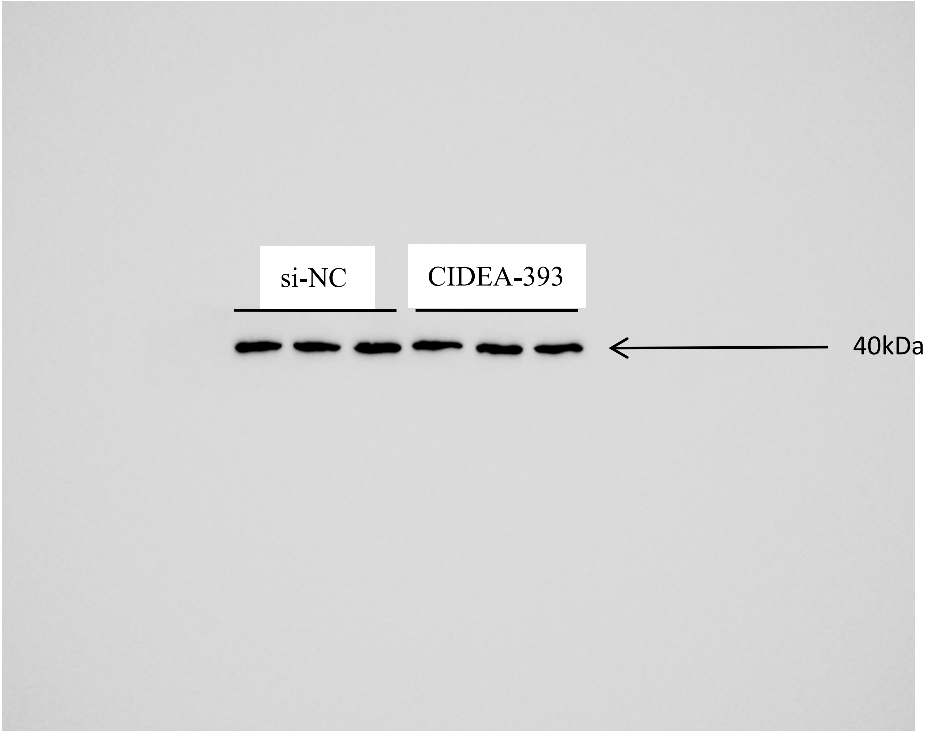

Figure S3
p-FAK

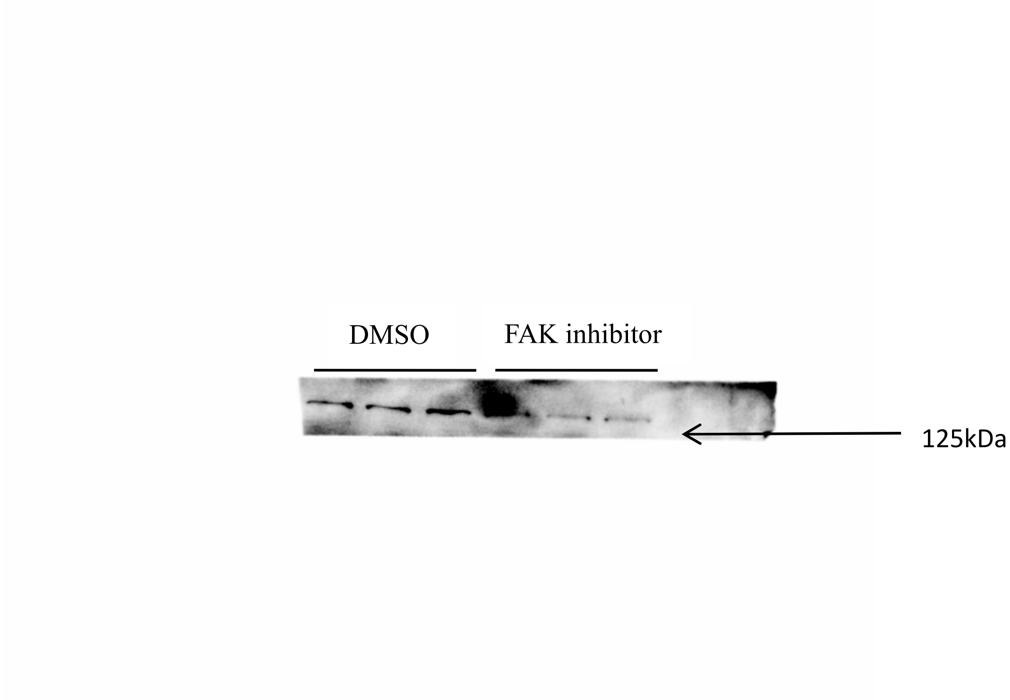

Figure S3
FAK

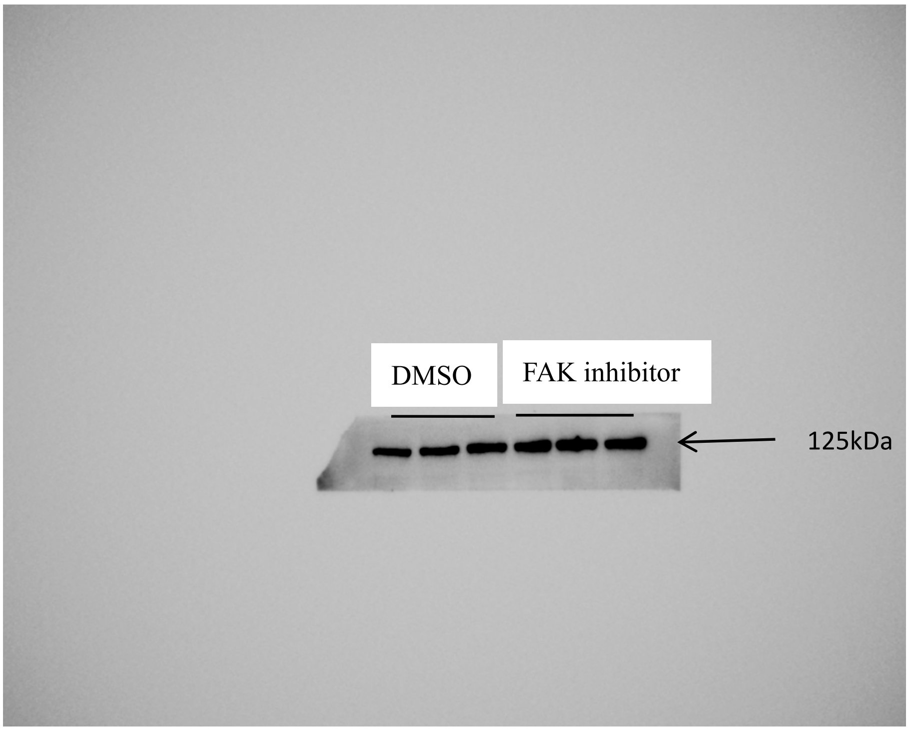

Figure S3

β -actin

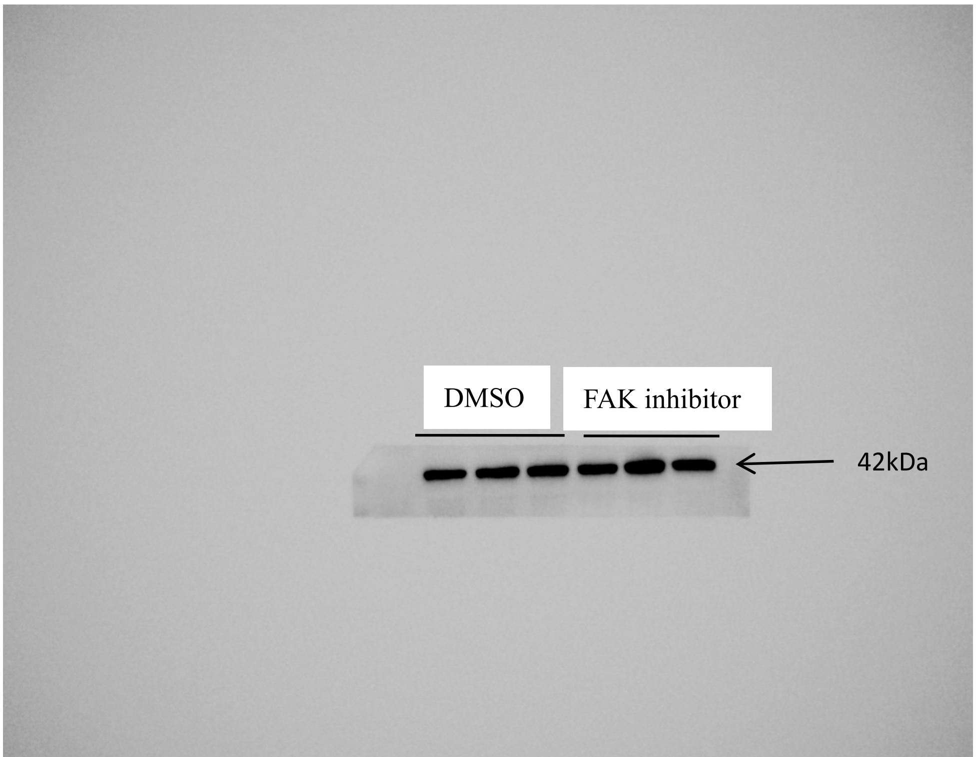

Supplement: Supplementary file 1 [file animals-15-02374-s001.zip › Original Western Blot Images.pdf]
